# Molecular Responses of Maize Shoot to a Plant Derived Smoke Solution

**DOI:** 10.3390/ijms20061319

**Published:** 2019-03-15

**Authors:** Muhammad Mudasar Aslam, Shafiq Rehman, Amana Khatoon, Muhammad Jamil, Hisateru Yamaguchi, Keisuke Hitachi, Kunihiro Tsuchida, Xinyue Li, Yukari Sunohara, Hiroshi Matsumoto, Setsuko Komatsu

**Affiliations:** 1 Department of Botany, Kohat University of Science and Technology, Kohat 26000, Pakistan; mudasar_kust@yahoo.com (M.M.A.); drshafiq@yahoo.com (S.R.); proteomics.sp@gmail.com (A.K.); 2 Faculty of Environmental and Information Sciences, Fukui University of Technology, Fukui 910-8505, Japan; 3 Faculty of Life and Environmental Sciences, University of Tsukuba, Tsukuba 305-8572, Japan; lixinyue108@gmail.com (X.L.); sunohara.yukari.gp@u.tsukuba.ac.jp (Y.S.); hmatsu@biol.tsukuba.ac.jp (H.M.); 4 Department of Biotechnology and Genetic Engineering, Kohat University of Science and Technology, Kohat 26000, Pakistan; Jamilkhattak@yahoo.com; 5 Institute for Comprehensive Medical Science, Fujita Health University, Toyoake 470-1192, Japan; hyama@fujita-hu.ac.jp (H.Y.); hkeisuke@fujita-hu.ac.jp (K.H.); tsuchida@fujita-hu.ac.jp (K.T.)

**Keywords:** proteomics, maize, plant-derived smoke, shoot

## Abstract

Plant-derived smoke has effects on plant growth. To find the molecular mechanism of plant-derived smoke on maize, a gel-free/label-free proteomic technique was used. The length of root and shoot were increased in maize by plant-derived smoke. Proteomic analysis revealed that 2000 ppm plant-derived smoke changed the abundance of 69 proteins in 4-days old maize shoot. Proteins in cytoplasm, chloroplast, and cell membrane were altered by plant-derived smoke. Catalytic, signaling, and nucleotide binding proteins were changed. Proteins related to sucrose synthase, nucleotides, signaling, and glutathione were significantly increased; however, cell wall, lipids, photosynthetic, and amino acid degradations related proteins were decreased. Based on proteomic and immunoblot analyses, ribulose-1,5-bisphosphate carboxylase/oxygenase (RuBisCO) was decreased; however, RuBisCO activase was not changed by plant-derived smoke in maize shoot. Ascorbate peroxidase was not affected; however, peroxiredoxin was decreased by plant-derived smoke. Furthermore, the results from enzyme-activity and mRNA-expression analyses confirmed regulation of ascorbate peroxidase and the peroxiredoxinin reactive oxygen scavenging system. These results suggest that increases in sucrose synthase, nucleotides, signaling, and glutathione related proteins combined with regulation of reactive oxygen species and their scavenging system in response to plant-derived smoke may improve maize growth.

## 1. Introduction

Maize is highly commercial crop, being a major source of food, feed, biofuel, and industrial products [1]. It is the most diverse crop analyzed at morphological and molecular levels [2]. Maize is an ideal crop for genomic studies because it exhibits a high level of genetic diversity and many structural variations [3]. Structural changes and genetic diversity play a key role in the morphology of maize [4]. Maize plant has a very large size genome with a complex organization [5,6]. These findings indicate the importance of maize genetic diversity for the manipulation of new resistant and high yielding varieties. 

Fire is documented as ecological factor in ecosystems, because many forest plant species’ life cycles depend on fire [7]. Fire products, which are heat, chemicals, ash, and smoke, have been widely identified as germination cues for different species from both fire-prone and fire-free ecosystems [8]. Plant-derived smoke contains active compounds to promote seed germination of crops [9]. The karrikins and cyanohydrins are identified as germination stimulants present in smoke [9]. These compounds have extensive implications for horticulture, weed control, conservation, and restoration [10]. Plant-derived smoke is a plant growth stimulant obtained from burning of wide variety of biotic sources including leaf, shoot, and straw [11]. These results indicate that plant-derived smoke and compounds isolated from smoke are vital stimulants for germination and plant growth.

Plant-derived smoke stimulated seed germination in 1200 plant species from more than 80 genera of different families [10] including crops [12], medicinal plants [13], and fruit [14]. The promotive effects of plant-derived smoke are independent of seed size, shape, and type [11]. In addition to stimulating seed germination, plant-derived smoke enhanced the seedling length/weight of different crops [15] and pollen germination/tube elongation of flowers belonging to different plant families [16]. Plant-derived smoke induces many changes in seeds, including sensitivity of seeds to phytohormones [17] and increased permeability by softening the seed coat [18]. These findings highlight the promotive effects of plant-derived smoke on plant morphology. 

Different physio-chemical contents of plants have been reported to be increased by plant-derived smoke solution [19]. This solution has stimulatory effects on photosynthesis in *Isatis indigotica* seedlings by enhancing carbon dioxide fixation, the transpiration rate, gaseous exchange, stomatal conductance, and photochemical activities [20]. In addition, total soluble proteins, chlorophyll *a*/*b,* total carotenoids, and total nitrogen contents were also increased in smoke treated rice seedlings [21]. Positive effects of plant-derived smoke were also observed on seed germinating enzyme activities in different grasses [22]. This smoke improved the plant-defense system by increasing flavonoids, tannins contents, and level of phenolics [23]. It is presumed that the stimulatory effects of plant-derived smoke are due to its close relation with plant growth regulator [24]. Despite these findings, the mechanism underlying these physiological changes remains unclear and needs an in-depth study to find plant-derived smoke effects on the physiological processes of plants.

Various morphological and physiological studies were conducted to analyze the effects of plant-derived smoke on plants growth; however, its mechanism of action on plant growth has not been investigated yet. The present study is focused on investigating the effects of plant-derived smoke on the initial seedling-stage of maize. Morphological analysis was performed on maize seedlings raised from smoke treated seeds. Based on these morphological results, a gel-free/label-free proteomic analysis was applied to assess the effects of plant-derived smoke solution on maize-shoot. It attempted to explore some clues about the response mechanism of plants towards plant-derived smoke solution at molecular level. In order to further validate and peep into the results at the molecular level, immunoblot, enzyme-activity, and mRNA-expression analyses were performed in a continuation of proteomic results.

## 2. Results

### 2.1. Morphological Effects of Plant-Derived Smoke on Maize Growth

**Figure 1:**
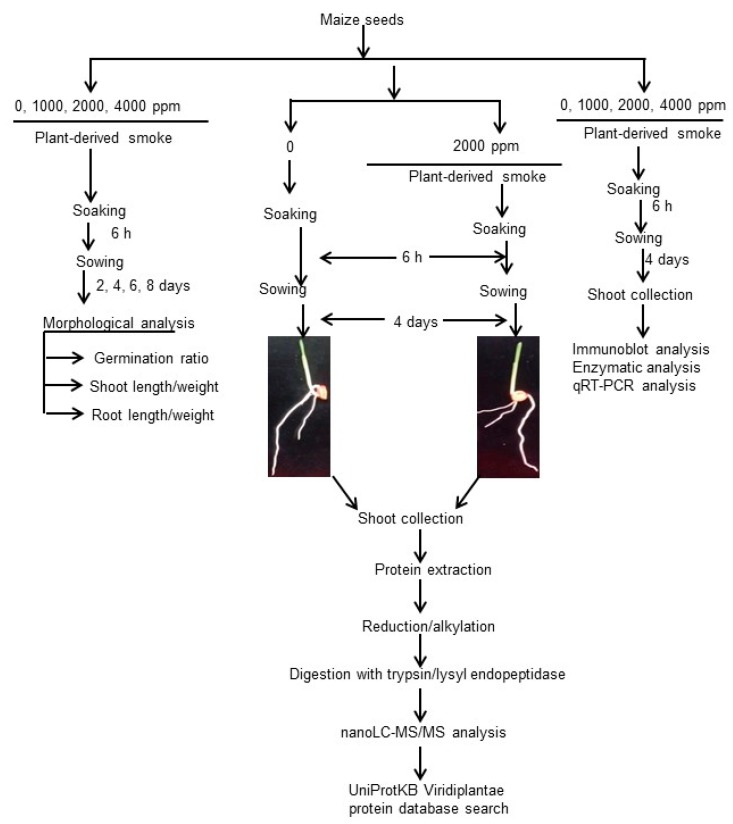
Experimental design for the effects of plant-derived smoke on maize growth. Maize seeds were soaked without or with 1000 ppm, 2000 ppm, and 4000 ppm plant-derived smoke for 6 h and grown in sand. For morphological analysis, germination ratio, shoot/root length, and weight were measured at 2, 4, 6, and 8 days. For proteomic analysis, maize seeds were soaked with 2000 ppm plant-derived smoke for 6 h. Shoot was collected after 4 days. Proteins were extracted, reduced, alkylated, digested, and analyzed by nano LC-MS/MS. For western blotting, maize seeds were soaked without or with 1000 ppm, 2000 ppm, and 4000 ppm plant-derived smoke for 6 h and grown in sand. Shoot was collected and proteins were extracted after 4 days for immune blotting analysis.

**Figure 2:**
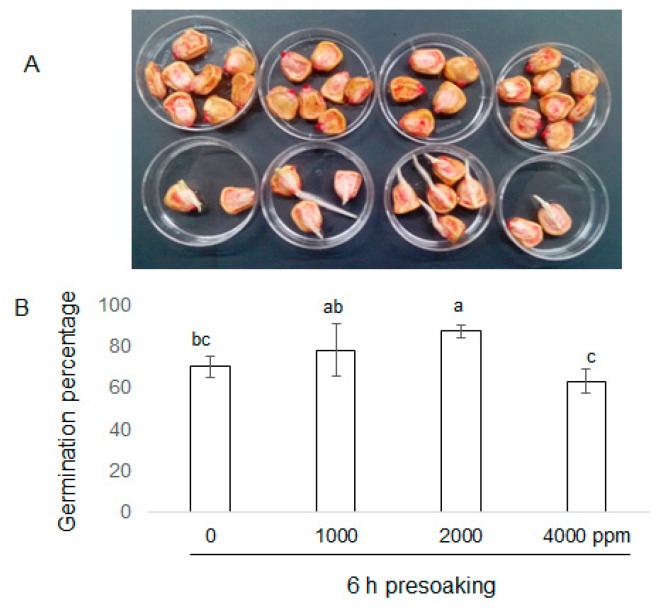
Germination percentage of maize seeds treated with plant-derived smoke. Maize seeds were soaked without or with 1000 ppm, 2000 ppm, and 4000 ppm plant-derived smoke for 6 h (**A**,**B**). The images in (**A**) correspond to data bars in (**B**). (**A**) The above row shows seeds, just after presoaking, while below row shows seeds, 2 days after presoaking. (**B**) Germination percentage was recorded after 2 days. The data are presented as the mean ± S.D. from 4 independent biological replicates. Different letters indicate that the change is significant as determined by one-way ANOVA followed by Tukey’s multiple comparison (*p* ˂ 0.05).

**Figure 3:**
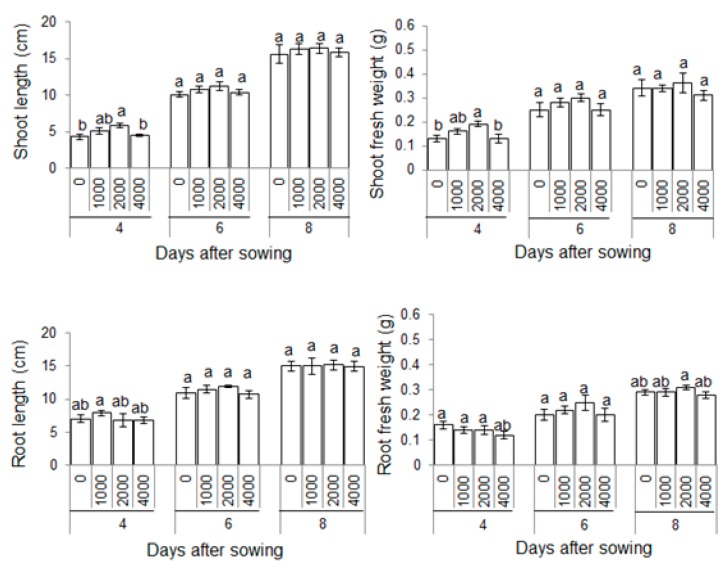
Morphological changes in maize treated with plant-derived smoke. Maize seeds were soaked without or with 1000 ppm, 2000 ppm, and 4000 ppm plant-derived smoke for 6 h and sown in sand for 4, 6, and 8 days. The length and fresh weight of shoot and root were measured. The data are shown as means ± S.D. from 4 independent biological replicates. Different letters indicate that the change is significant as determined by one-way ANOVA followed by Tukey’s multiple comparison (*p* ˂ 0.05).

To investigate the effects of plant-derived smoke on maize growth, morphological analysis was performed. Seeds were soaked without or with 1000 ppm, 2000 ppm, and 4000 ppm plant-derived smoke for 6 h. Length and fresh weight of shoot and root were measured for 4, 6, and 8 days after sowing (Figure 1). The seed germination percentage was significantly increased by 2000 ppm plant-derived smoke as compared to control while 4000 ppm plant-derived smoke did not affect germination percentage (Figure 2). Length and fresh weight of shoot were increased by treatments of 1000 ppm and 2000 ppm plant-derived smoke 4 days after sowing (Figure 3). The length of the root was increased more by the 1000 ppm treatment than by the control as indicated in Figure 3; and the increase was not significant with the 2000 ppm treatment.

The fresh weight of the root was not significantly changed by 1000 ppm or 2000 ppm treatment compared to untreated plant (Figure 3). The length and fresh weight of root were not affected by plant-derived smoke concentrations 6 days after sowing. Treatment with 4000 ppm plant-derived smoke did not affect THE length and fresh weight of shoot and root compared to an untreated plant. 

### 2.2. Functional Classification of Differentially Abundant Proteins in Maize Treated with Plant-Derived Smoke

Maize seeds were treated with 2000 ppm plant derived smoke solution and were grown for 4 days. Proteins were extracted from the 4-days old maize shoot and proteomic response was then investigated. The criteria for significantly changed proteins were 2 or more than 2 matched peptides with a *p*-value less than 0.05. Gel-free/label-free proteomic analysis revealed that a total of 69 proteins were significantly changed by 2000 ppm plant-derived smoke (Table 1). Results showed that in the biological process category, metabolic related proteins were prominently changed as compared to other categories (Figure S1). For the cellular compartment, cytoplasm localized proteins were significantly changed by plant-derived smoke (Figure S1). For the molecular function, the proteins with catalytic activity were more significantly changed than the other categories (Figure S1).

**Figure 4:**
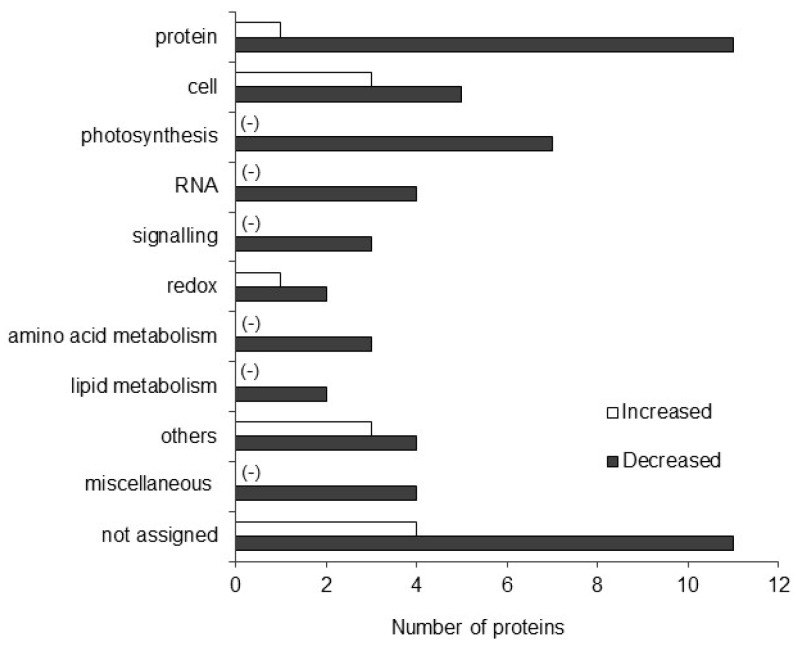
Categorization of identified proteins on the basis of their function in maize shoot in response to plant derived smoke solution. Maize seeds were treated without or with 2000 ppm plant-derived smoke solution for 6 h and grown for 4 days. Proteins were extracted from shoot and identified using a gel-free/label-free proteomic technique. Mapman bin code software was used to categories all the identified 69 proteins. Categories containing zero proteins are marked with (−). Abbreviations: protein, protein synthesis/degradation/ post-translational modification/targeting/folding; cell, cell division/organization/vesicle transport; RNA, RNA processing/ transcription/binding; Redox, redox homeostasis; The “Others” category includes proteins related to N-metabolism, cellular homeostasis, response to stimulus, cytoplasm, fermentation, DNA, nucleotide binding, and metal ion binding.

To determine the function of proteins in response to the plant-derived smoke solution, functional classification of identified proteins was performed using MapMan bin codes (Figure 4). It was found that 1 and 11 proteins involved in protein synthesis, degradation, post-translational modification, targeting and folding were increased and decreased, respectively, in response to plant-derived smoke solution. Plant-derived smoke treatment increased one and decreased two proteins related to redox homeostasis. Three proteins related to signaling and 4 transport related proteins were also increased, while RNA, amino acid metabolism and lipid metabolism related proteins were decreased in maize shoot in response to the plant-derived smoke solution (Figure 4).

### 2.3. Pathway Analysis of Identified Proteins in Maize Treated with Plant-Derived Smoke

The plant-derived smoke treatment significantly changed some proteins. To visualize these proteomic results in the context of pathways and processes, MapMan software was used (Figure S2). Proteins associated with starch/sucrose, glutathione and nucleotide biosynthesis were significantly increased; while, ascorbate, photosynthetic, lipids, and cell wall related proteins were decreased in response to plant-derived smoke (Figure S2).

### 2.4. Immuno-Blot Analysis of Proteins Involved in Redox Homeostasis and Photosynthesis 

**Figure 5:**
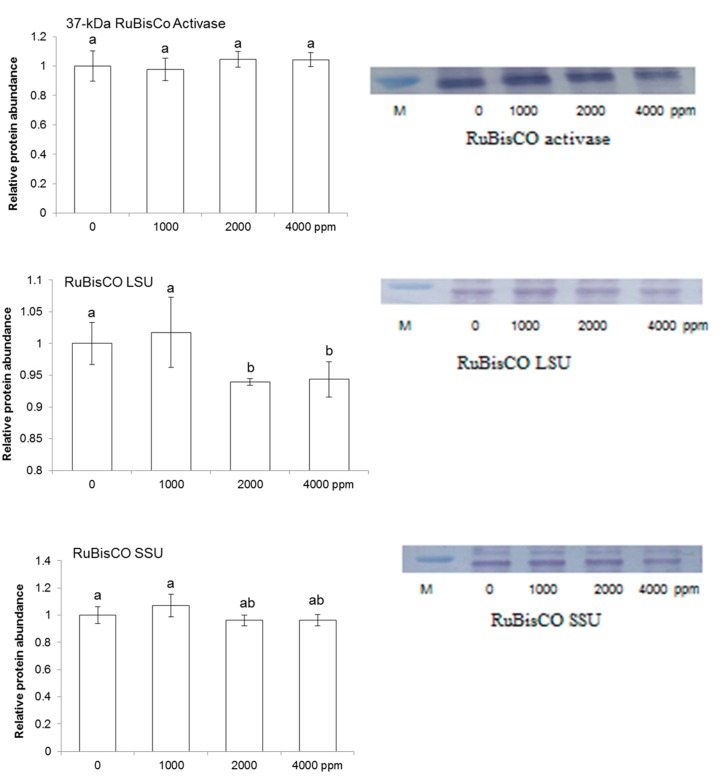
Effect of plant-derived smoke on RuBisCO subunits and activase enzymes in maize. Maize seeds were treated without or with 1000, 2000, and 4000 ppm plant-derived smoke for 6 h and sown in sand. Proteins were extracted from 4-days old maize shoot. ImageJ software was used to determine relative proteins intensities. Data are shown as means ± S.D. Different alphabets showing the statistical level of significance as determined by one-way ANOVA followed by Tukey’s multiple comparison (*p* ˂ 0.05). In images, ‘M’ is used for marker.

**Figure 6:**
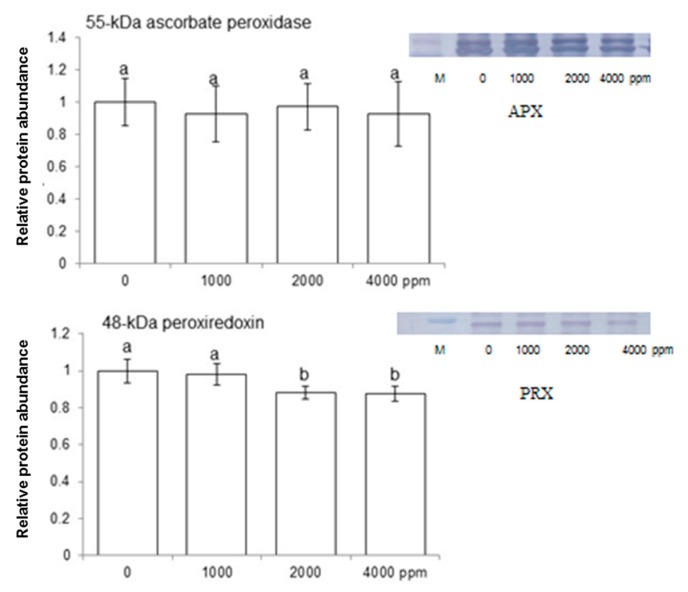
Effect of plant-derived smoke on redox-homeostasis related proteins in maize. Maize seeds were treated without or with 1000, 2000, and 4000 ppm plant-derived smoke for 6 h and grown for 4 days. Proteins extracted from shoot were separated by SDS-polyacrylamide gel electrophoresis and transferred onto polyvinylidine difluoride membranes. The membranes were incubated with anti-RuBisCO large/small subunits and anti-RuBisCO activase antibodies. The relative intensities of bands were calculated using ImageJ software. Data are shown as means ± S.D. from 3 independent biological replicates. Different letters indicate that the change is statistically significant as determined by one-way ANOVA followed by Tukey’s multiple comparison (*p* ˂ 0.05). In images, “M” is used for marker.

To find out abundance level of large and small RuBisCO subunits, RuBisCO activase, ascorbate peroxidase, and peroxiredoxin in maize shoot, immunoblot analysis was performed. Maize seeds were treated without or with 1000 ppm, 2000 ppm, and 4000 ppm plant-derived smoke and proteins were extracted from shoot after 4 days of sowing (Figure 1). Plant-derived smoke did not affect the abundance of 37-kDa RuBisCO activase while decreasing the abundance of RuBisCO large subunit in shoot of 4-day-old plants treated by 2000 ppm and 4000 ppm (Figure 5). Abundance of 55 kDa ascorbate peroxidase was not affected by different treatments of plant-derived smoke; while, 2000 ppm and 4000 ppm plant-derived smoke very slightly decreased the abundance of 37-kDa peroxiredoxin compared to 1000 ppm and the control (Figure 6).

### 2.5. Effect of Plant-Derived Smoke on Enzymatic Activities and Gene Expression of Maize

**Figure 7:**
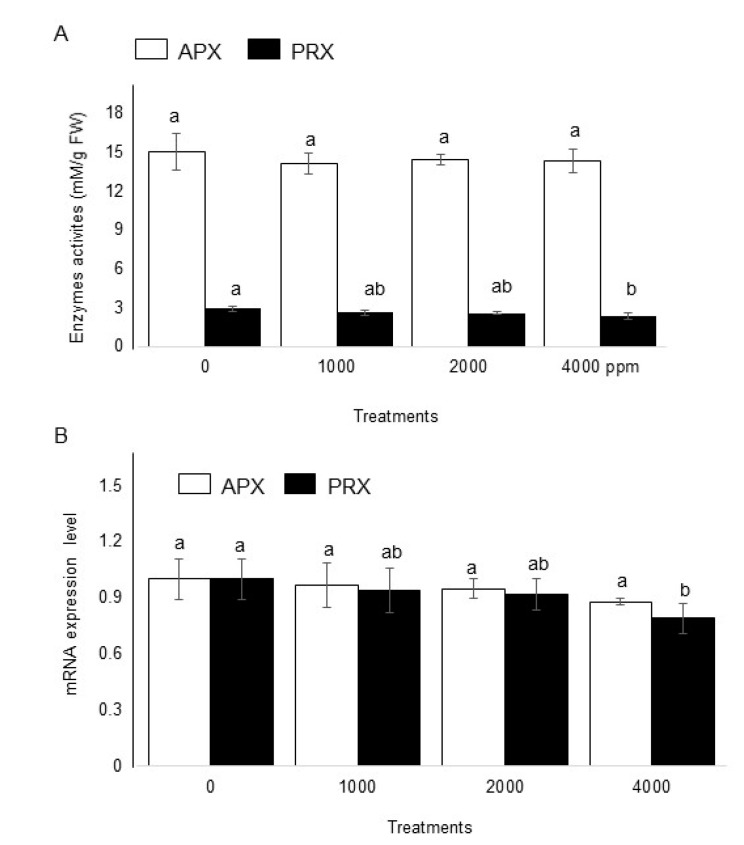
Effect of plant-derived smoke on enzymatic analysis and expression level of ascorbate peroxidase and peroxiredoxin gene in maize shoot. Maize seeds were treated without or with 1000 ppm, 2000 ppm, and 4000 ppm plant-derived smoke for 6 h and sown. Activities of ascorbate peroxidase (APX) and peroxiredoxin (PRX) were measured in 4-day old maize shoot (**A**). For gene expression level, RNAs extracted from maize shoot at 3rd day of treatment were analyzed by qRT-PCR (**B**). Relative mRNA abundances of ascorbate peroxidase and peroxiredoxin were normalized against 18S rRNA abundance. The data are shown as means ± S.D. from 4 independent biological replicates. White and black bars indicate ascorbate peroxidase (APX) and peroxiredoxin (PRX), respectively. Different letters indicate that the change is significant as determined by one-way ANOVA followed by Tukey’s multiple comparison (*p* ˂ 0.05).

To investigate the effects of plant-derived smoke on ROS scavenging enzymes, enzymatic analysis was performed. Seeds were soaked in without or with 1000 ppm, 2000 ppm, and 4000 ppm plant-derived smoke for 6 h and maize shoot was collected 4 days after sowing (Figure 1). Results showed that 1000 ppm, 2000 ppm, and 4000 ppm plant-derived smoke did not change activity of ascorbate peroxidase as compared to the control (Figure 7A). On the other hand, treatments 1000 ppm and 2000 ppm and 4000 ppm slightly, but significantly affected the activity of peroxiredoxin with a significant decrease compared to the control (Figure 7A).

To check the specific expression pattern of genes encoding ascorbate peroxidase and peroxiredoxin, their mRNA levels in maize shoot were analyzed. Shoot total RNA was extracted, and qRT-PCR was performed. The 18S rRNA was used as an internal control on qRT-PCR. Results revealed that 1000 ppm, 2000 ppm and 4000 ppm plant-derived smoke did not significantly affect the expression level of ascorbate peroxidase (Figure 7B); however, the expression level of peroxiredoxin was regulated with decreased expression at the transcript level in response to plant-derived smoke (Figure 7B).

## 3. Discussion

### 3.1. Effect of Plant-Derived Smoke on Morphology of Maize 

Plant-derived smoke increased seed germination in different plant species [25]. Soós et al. [26] reported that plant-derived smoke enhanced the post-germination growth of maize. In this study, 2000 ppm plant-derived smoke significantly increased the seed germination percentage; furthermore, shoot/root length/fresh weight were also increased in maize. Aslam et al. [27] reported that plant-derived smoke increased the germination percentage, shoot/root length, and fresh weight of maize. Furthermore, plant-derived smoke treated maize developed vigorous seedling and fresh weight [28]. Abu et al. [29] reported that priming seeds in low concentrations of plant-derived smoke increased the germination percentage and seedling length in wild rye. On the other hand, high concentrations of plant-derived smoke reduced germination and shoot/root growth in lettuce [30]. In this study, 4000 ppm plant-derived smoke did not affect maize seed germination and shoot/root length/fresh weight (Figure 2 and Figure 3). This finding is similar to previous results revealing that plant-derived smoke inhibited seed germination percentage at higher concentrations.

Plants treated with 2000 ppm plant-derived smoke and 10^−8^ M karrikins produced vigorous shoot and increased leaf area in *Eucomis autumnalis* [31]. The vigorous shoot and high leaf area enhanced photosynthetic activities in plants [31]. Flematti et al. [32] reported that plant-derived smoke containing karrikins enhanced germination percentage and shoot length/fresh weight in *Solanum orbiculatum*. Plant-derived smoke improved the shoot length and fresh weight in several plants, including acacia [33], onion [34], and milk thistle [35]. Our results are in accordance with previous findings suggesting that plant-derived smoke increases shoot length and fresh weight. Based on these results, 4-days old shoot raised from maize seeds treated by 2000 ppm plant-derived smoke was selected for gel-free/label free proteomic analysis.

### 3.2. Positive Effect of Plant-Derived Smoke on Cytoskeleton Related Proteins in Maize

With treatment of plant-derived smoke, the fold change of cytoskeleton related proteins such as profilin and actin was significantly increased compared to protein abundance of untreated plant (Table 1). The plant cytoskeleton plays an important role in many biological processes, including cell division, expansion, organogenesis, tip growth, and intracellular signaling [36]. The actin cytoskeleton proteins are concerned with the establishment and maintenance of cell polarity, and responses to numerous environmental stimuli [37]. Profilin promoted F-actin elongation which played an important role in the mobility and cell contraction during cell division [38]. Profilins are multifunctional proteins according to their abundance and locations [39]. Furthermore, profilin is associated with plasma membrane during development of microspores, pollens, and playing vital role in signal transduction [40]. The present results confirmed that seed priming in plant-derived smoke promoted shoot cytoskeleton proteins, which are necessary for cell division, signaling, and pollens development.

### 3.3. Effect of Plant-Derived Smoke on Photosynthetic Proteins in Maize

Plant derived smoked changed 8 photosynthesis related proteins with different regulation. RuBisCO is the most abundant protein on earth playing key role in photosynthesis having CO_2_ fixation function [41]. Strong reduction was observed in activity of RuBisCO under drought stress [42]. Khodadadi et al. [43] observed that the activity of RuBisCO was reduced under drought stress. Another study reported that RuBisCO is responsive to stresses and the rapid loss of its activity caused by drought in sunflower [44]. Beside drought, cold stress also reduced the activity of RuBisCO, suggesting damage to the chloroplasts and a decrease in the rate of photosynthesis under these conditions. In plant-smoke derived compounds, karrikins also decreased RuBisCO in *Arabidopsis* [45]. The present immunoblot result revealed that RuBisCO activase is not affected by plant-derived smoke; however, a RuBisCO large subunit was decreased by plant-derived smoke. It may be possible that plant-derived smoke plays an inhibitory role at higher concentrations. It is also possible that this short period of presoaking in plant-derived smoke is not enough to activate the RuBisCO activase enzyme.

### 3.4. Effect of Plant-Derived Smoke on Scavenging Activity through the Ascorbate/Glutathione Pathway in Maize

Ascorbate and glutathione are main antioxidants in the leaves of maize [46]. The fundamental role of these compounds is linked to scavenging a broad range of reactive oxygen species generated under stress conditions in plants [47]. It participates in scavenging of hydrogen peroxide [48]. Selote and Chopra [49] reported that drought stress increased the amount of glutathione in rice. It is reported that ascorbate peroxidase was decreased under flooding conditions [50]. Present results revealed that ascorbate is decreased while glutathione is increased at the proteomic level. These results suggest that plant-derived smoke seed pretreatment may have positive effects on glutathione pool in the maize seedlings that might be helpful in achieving better performance during stress responses.

### 3.5. Effect of Plant-Derived Smoke on Enzymatic Activities of Maize

Ascorbate peroxidase and peroxiredoxin are important enzymes that are present in the plant kingdom [51]. The exposure of plants to unfavorable environmental conditions increases the production of reactive oxygen species such as hydrogen peroxide, and hydroxyl radical [52]. The reactive oxygen species detoxification process in plants is essential and occurs due to different enzymes like ascorbate peroxidase and peroxiredoxin in plant cells and their organelles [52]. Ascorbate peroxidase utilizes ascorbate as a specific electron donor to reduce hydrogen peroxide to water [53]. Experimental evidence has proven that during the metabolism process, antioxidant enzymes are also produced to balance metabolism [54]. On the other hand, peroxiredoxins constitute the most recently identified group of hydrogen peroxide -decomposing antioxidant enzymes [55]. It is reported that plant-derived smoke decreased scavenging enzymes activity in maize [56]. Another redox homeostasis enzymes, thioredoxins (Trxs) was also affected by plant-derived smoke treatments. It is a small and widely distributed protein with a conserved active site, which controls the redox status of target proteins through thiol-disulfide exchange reactions [57]. In plants, it has a fundamental role in a number of cellular processes, including seed germination, carbon assimilation, lipid metabolism, hormone metabolism, redox signaling, and stress response [57]. The present results are in accordance with the previous reports and showed that plant-derived smoke did not significantly affect the activities of ascorbate peroxidase and that peroxiredoxin activities were decreased, which revealed that plant-derived smoke is a growth promoter and stress suppressor, which decreases the production of stress responsive hormones. 

Antioxidants are mostly expressed to cope with stressed situations and their expression also has positive effects on the activation of enzymes related to plant growth [58,59]. In the present study, plant-derived smoke is behaving as a growth promoter and thus did not affected ascorbate peroxidase enzyme levels (Figure 7A). Auxins might not affect or could promote increases in the activity of antioxidant enzymes regulating ROS levels, which could be associated with the activation of embryo/organogenesis [60]. It might be suggested from the results that treatment with plant-derived smoke could markedly enhance the self-capacity of defense against oxidative damage in normal growth conditions, thus not affecting the production of antioxidant enzymes in maize seedlings significantly.

### 3.6. Effect of Plant-Derived Smoke on Expression of Ascorbate Peroxidase and Peroxiredoxin in Maize

Plant-derived smoke did not affect ascorbate peroxidase gene expression level, whereas gene expression of peroxiredoxin was decreased at higher concentrations (Figure 7B). Ascorbate peroxidase is an important enzyme of plant scavenging system. It uses ascorbate as a specific electron donor for the conversion of hydrogen peroxide into water [61]. Besides this, it also improves the stress resistance capacity in plants against various stresses [62,63]. Ascorbate peroxidase also plays a key role in balancing the homeostasis of ascorbate and glutathione, and maintaining high photosynthetic rate in unfavorable conditions [64]. In addition, ascorbate is involved in other functions such as plant growth, gene regulation, modulation of some enzymes, and redox regulation of membrane-bound antioxidant compounds [65]. The present results revealed that ascorbate peroxidase related gene was not significantly altered in maize seedlings due to plant-derived smoke. The present results are consistent with immunoblotting results showing no effect on the abundance of ascorbate peroxidase. Decreased peroxiredoxin abundance in maize shoot in response to plant derived smoke reflects the resource economy phenomena of all living organisms, including plants. Peroxiredoxin might be present in maize plants, taking part in plant defense system against stresses. There is close interaction between plant-derived smoke and plant growth hormones [24]. It is also possible that plant immune systems might be strengthened by plant-derived smoke, resulting in a decreased level of peroxiredoxin level so as to economize plant resources. These results are in agreement with El-Gaied et al. [59] who clearly demonstrated the decreased antioxidant enzymes level in the tomato plant in response to plant growth promoting hormones.

## 4. Materials and Methods

### 4.1. Preparation of Plant-Derived Smoke Solution

Smoke solution was prepared from aerial semi dried parts of *Cymbopogon jawarncusa* [66]. Dry plant material weighing 333 g was taken and placed in a burner [67]. An electric heater was adjusted beneath the burner until all the plant material was converted into ash. Smoke was bubbled though 1 L of distilled water, resulting into 1 L of concentrated plant-derived smoke solution. It was further diluted to 1000 ppm, 2000 ppm, and 4000 ppm and used for seed treatment in the experiment. The seeds treated with distilled water were used as the control.

### 4.2. Plant Material and Treatment

Seeds of maize (*Zea mays* L. cv. Azam) were sterilized with 3% sodium hypochlorite solution. The sterilized seeds were primed with 1000 ppm, 2000 ppm, and 4000 ppm plant-derived smoke for 6 h, and then sown in seedling case (150 mm × 60 mm × 100 mm) supplied with water. The seeds treated with distilled water were used as control. Maize was grown in growth chamber illuminated with white fluorescent light (160 μmol·m^−2^·s^−1^, 16 h light period/day) at 25 °C with 60% humidity. Germination percentage was recorded after two days of sowing. Fifteen seeds were sown for each treatment and 4 independent replicates were used for morphological analysis. 

For proteomics and further investigation through immunoblot analysis, enzymatic analysis and qRT-PCR, maize seeds (*Zea mays* L. cv. Honey Bantam) were sterilized using 3% sodium hypochlorite solution, soaked in 2000 ppm plant-derived smoke for 6 h and grown in 450 mL silica sands with water in seedling case (150 mm × 60 mm × 100 mm). The seeds treated with distilled water were used as control. Conditions in growth chamber were illumination with white fluorescent light (160 μmol·m^−2^·s^−1^, 16 h light period/day) at 25 °C with 60% humidity. Shoot was collected on the 4th day after sowing from 3 biological replicates. Shoot raised from the seeds treated with distilled water served as control. Biological replicates mean that maize was sown on different days.

### 4.3. Protein Extraction

A portion (300 mg) of maize shoot was cut into small pieces and ground 60 times in 2 mL tube. It was ground 30 times after adding 50 µL of lysis buffer containing 7 M urea, 2 M thiourea, 5% CHAPS, and 2 mM tributylphosphine. Furthermore, 50 µL of lysis buffer was added and ground for 30 times. Suspension was incubated for 2 min at 25 °C and centrifuged at 15,000× *g* for 2 min at 25 °C. Afterwards, the filter cartridge was removed and supernatant was collected as total proteins.

### 4.4. Protein Enrichment, Reduction, Alkylation, and Digestion

Extracted proteins (100 µg) in lysis buffer were adjusted to a final volume of 100 µL. Methanol (400 µL) was added to each sample and mixed before the addition of 100 µL of chloroform and 300 µL of water. After mixing and centrifugation at 20,000× *g* for 10 min to achieve phase separation, the upper phase was discarded and 300 µL of methanol was added to the lower phase, and then centrifuged at 20,000× *g* for 10 min. The pellet was collected as the soluble fraction [68]. 

Proteins were resuspended in 50 mM NH_4_HCO_3_, reduced with 50 mM dithiothreitol for 30 min at 56 °C, and alkylated with 50 mM iodoacetamide for 30 min at 37 °C in the dark. Alkylated proteins were digested with trypsin and lysyl endopeptidase (Wako, Osaka, Japan) at a 1:100 enzyme/protein ratio for 16 h at 37 °C. Peptides were desalted with Mono Spin C18 Column (GL Sciences, Tokyo, Japan). Peptides were acidified with formic acid (pH < 3) and analyzed by nano-liquid chromatography (LC) mass spectrometry (MS)/MS.

### 4.5. Measurement of Protein and Peptide Concentrations

The method of Bradford [69] was used to determine the protein concentration with bovine serum albumin used as the standard. 

### 4.6. Protein Identification Using NanoLC-MS/MS

The peptides were loaded onto the LC system (EASY-nLC 1000; Thermo Fisher Scientific, San Jose, CA, USA) equipped with a trap column (Acclaim PepMap 100 C18 LC column, 3 µm, 75 µm ID × 20 mm; Thermo Fisher Scientific) equilibrated with 0.1% formic acid and eluted with a linear acetonitrile gradient (0–35%) in 0.1% formic acid at a flow rate of 300 nL/min. The eluted peptides were loaded and separated on the column (Easy-Spray C18 LC column, 3 µm, 75 µm ID × 150 mm; Thermo Fisher Scientific) with a spray voltage of 2 kV (Ion Transfer Tube temperature: 275 °C). The peptide ions were detected using MS (Orbitrap Fusion EDT MS; Thermo Fisher Scientific) in the data-dependent acquisition mode with the installed Xcalibur software (version 4.0; Thermo Fisher Scientific). Full-scan mass spectra were acquired in the MS over 375–1500 *m*/*z* with resolution of 120,000. The most intense precursor ions were selected for collision-induced fragmentation in the linear ion trap at a normalized collision energy of 35%. Dynamic exclusion was employed within 90 s to prevent repetitive selection of peptides [70].

### 4.7. MS Data Analysis

The MS/MS searches were carried out using the Mascot (version 2.6.1, Matrix Science, London, UK) and SEQUEST HT search algorithms against the UniProtKB Viridiplantae protein database (2017-07) using Proteome Discoverer 2.1 (version 2.1.1.21; Thermo Fisher Scientific). The workflow for both algorithms included a spectrum selector, Mascot, SEQUEST HT search nodes, percolator, ptmTS, event detector, and precursor ion area detector nodes. Oxidation of methionine was set as a variable modification and carbamidomethylation of cysteine was set as a fixed modification. MS and MS/MS mass tolerance were set to 10 ppm and 0.6 Da, respectively. Trypsin was specified as the protease and a maximum of one missed cleavage was allowed. Target-decoy database searches used for calculation of false discovery rate (FDR) and for peptide identification FDR was set at 1%. Label-free quantification was also performed with Proteome Discoverer 2.1 using precursor ions area detector nodes.

### 4.8. Differential Analysis of Proteins Using MS Data

For differential analysis of the relative abundance of peptides and proteins between samples, the freely software PERSEUS (version 1.6.0.7) [71] was used. Proteins and peptides intensities were transferred into log2 scale. Three biological replicates of each sample were grouped and a minimum of 3 valid values was required in at least one group. Normalization of the intensities was performed to subtract the median of each sample. Missing values were imputed based on a normal distribution (width = 0.3, down-shift = 2.2). Significance was assessed using student’s *t*-test analysis. Accession codes is as follows: For MS data, RAW data, peak lists and result files have been deposited in the ProteomeXchange Consortium [72] via the jPOST [73] partner repository under data-set identifiers PXD008315.

### 4.9. Functional Categorization

The protein sequences of the differentially changed proteins, based on the Lan10 strain, were subjected to a BLAST query against the Ami gene ontology database (http://amigo1.geneontology.org/cgi-bin/amigo/blast.cgi). The corresponding Ami gene ontology terms were extracted from the most homologous proteins using a Perl program. The Ami gene ontology database annotation results were plotted by the Web Gene Ontology Annotation Plot (http://wego.genomics.org.cn/) tool by uploading compiled Web Gene Ontology Annotation Plot native format files containing the obtained Ami gene ontology terms. Functional categorization of identified proteins was performed using MapMan bin codes (http://mapman.gabipd.org/) [74]. Visualization of protein abundance was performed using MapMan software (version 3.6.0 RC1, http://mapman.gabipd.org/web/guest/mapman) [75]. The software and mapping files of Gmax_109_peptide were also downloaded from the MapMan website. 

### 4.10. Immunoblotting Analysis

Maize shoot (100 mg) sample was ground in an SDS-sample buffer consisting of 60 mM Tris-HCl (pH 6.8), 2% SDS, 10% glycerol, and 5% 2-mercaptoethanol using mortar and pestle [76]. The obtained mixture was centrifuged 2 times at 15,000× *g* for 10 min and protein was collected as a supernatant. SDS-polyacrylamide gel electrophoresis was used to separate protein (10 µg) in SDS-sample buffer. The separated proteins were shifted to polyvinylidene difluoride membrane using a semi-dry transfer blotter. A buffer containing 137 mM NaCl, 20 mM Tris-HCl (pH 7.5), 0.1% Tween-20, and a blocking solution (Wako) was used to block the blotted membrane for 1 h. Afterwards, different diluted (1:1000) anti-ascorbate peroxidase antibody [77], anti-peroxiredoxin antibody [78], anti-ribulose-1,5-bisphosphate carboxylase/oxygenase (RuBisCO) large and small subunits antibodies [79], and anti-RuBisCO activase antibody [80] was used to incubate the membrane for 1 h. The membrane was washed 3 times with buffer containing 137 mM NaCl, 20 mM Tris-HCl (pH 7.5), and 0.1% Tween-20 and treated for 1 h with anti-rabbit IgG conjugated with horseradish peroxidase (Bio-Rad, Hercules, CA, USA) as secondary antibody. The membrane was incubated with TMB membrane peroxidase substrate system (KPL, Sylacauga, AL, USA). ImageJ software (version 1.46, https://imagej.nih.gov/ij/) was used to calculate the relative intensities of bands.

### 4.11. Enzymatic Analysis

For ascorbate peroxidase analysis, a sample (1 g) of maize shoot was ground in pestle and mortar with liquid nitrogen. This grinded mixture was homogenized in 50 mM potassium phosphate buffer (pH 7.0) containing 0.5 mM Ascorbic acid, 0.1 mM EDTA and 0.1 mM hydrogen peroxide [81]. The hydrogen peroxide-dependent oxidation of Ascorbic acid was followed by monitoring the decrease in absorbance at 290 nm assuming an absorption coefficient of 2.8 mM^−1^·cm^−1^. For peroxiredoxin analysis, the assay contains 100 mM potassium phosphate-buffer (pH 7.0), 0.3–3 µM peroxiredoxin, 100 µM hydrogen peroxide in a total volume of 1000 µL. The reaction was stopped with 800 µL oftrichloroacetic acid (12.5%) to an aliquot of 50 µL of assay solution. After the addition of 200 µL, 10 mM Fe (NH_4_)_2_(SO_4_)_2_ and 100 µL of 2.5 M KSCN, the absorbance at 480 nm was measured to quantify the hydrogen peroxide contents of the solution, and hydrogen peroxide reduction rates were calculated [82].

### 4.12. RNA Extraction and Reverse Transcription Polymerase Chain Reaction Analysis

A total of 100 mg maize shoot was ground in mortar and pestle using liquid nitrogen. Total RNAs was extracted by RNeasy plant mini kit (Qiagen, Valencia, CA, USA) from maize shoot powder and treated with RNase-free DNase I during extraction. cDNA was synthesized from the extracted RNAs by using RevertAid first strand cDNA synthesis Kit (Thermo Scientific) in reverse transcription polymerase chain reaction (qRT-PCR). Fast Real Time PCR system (7900HT; Applied Biosystems, Foster City, CA, USA) was used to perform a qRT-PCR reaction at the following conditions; 95 °C for 600 s, followed by 35 cycles of 95 °C for 15 s and 60 °C for 60 s. As an internal control, 18S rRNA was used to normalize the gene expression. For normalization of gene expression, 18S rRNA was used as an internal control. For quantification of specific gene, primers were designed for regions of interest using NCBI tool (primer blast) and Primer 3 online bioinformatics tools. Quantitative variation between different samples was calculated by the relative quantification method (2^−ΔΔ*C*t^) [83].

### 4.13. Statistical Analysis

Data were analyzed by one-way ANOVA followed by Tukey’s multiple comparison among multiple groups using SPSS (version 22.0; IBM, Armonk, New York, USA). A *p*-value of less than 0.05 was considered to be statistically significant.

## 5. Conclusions

Gel-free/label-free proteomic technique was used to examine the effects of plant-derived smoke on maize growth after 6 h presoaking. The main results of this study are as follows: (i) Plant-derived smoke increased seed germination and seedling length/ fresh weight at low concentrations; (ii) Nucleotide, starch degradation, and glutathione related proteins were increased; (iii) Protein synthesis/degradation and cell division/organization proteins were changed; (iv) Cell wall, lipids, photosynthetic, and amino acid degradations related proteins ware decreased; (v) plant-derived smoke increased cytoskeleton proteins in maize; (vi) plant-derived smoke did not affect the activity of ascorbate peroxidase and decreased the activity of peroxiredoxin; (vii) gene expression level of peroxiredoxin was altered by plant-derived smoke. These results suggest that plant-derived smoke affects the proteins related to metabolic processes while inhibiting proteins related with lipids, proteins, and cell wall. Furthermore plant-derived smoke regulates the reactive oxygen species and their scavenging system. Although various studies have been conducted demonstrating the promoting effects of plant derived smoke solution on different growth parameters of plants, the molecular response of plant to plant derived smoke solution remained unknown. This study was carried out to fill the gap between various physiological processes regulated by a plant derived smoke solution and the possible mechanism of action behind it.

## Figures and Tables

**Table 1:** List of proteins altered by plant-derived smoke solution in maize shoot.

No	Accession	Description	Fold Change	Functional Category	Biological Process	Cellular Component	Molecular Function
1	B4FTX3	Profilin	7.22	Cell	not assigned	cell part	not assigned
2	B9RWF4	Elongation factor 1-alpha	6.79	Protein	not assigned	cell part	binding
3	A0A1D6BQY9	Tubulin beta chain	4.91	Cell	not assigned	not assigned	not assigned
4	C5XR12	Uncharacterized protein	4.89	Not assigned	not assigned	not assigned	catalytic activity
5	K7TFA7	Aldo/keto reductase family protein	4.17	Redox	biosynthetic process	cell part	catalytic activity
6	C9VWQ3	Actin	3.99	Cell	not assigned	not assigned	not assigned
7	A0A1D6DZR9	AICARFT/IMPCHase bienzyme	3.11	Nucleotide metabolism	not assigned	not assigned	not assigned
8	C5YEF7	Uncharacterized protein	2.91	Not assigned	not assigned	cell part	adenyl nucleotide binding
9	B6T289	Glucan endo-1,3-β-glucosidase 7	2.55	Not assigned	carbohydrate metabolic process	not assigned	catalytic activity
10	A0A1B6QAM8	Uncharacterized protein	2.36	Major CHO metabolism	not assigned	not assigned	not assigned
11	B4FTR5	Uncharacterized protein	1.95	Not assigned	cell communication	cell part	not assigned
12	C8ZK16	Malic enzyme	1.37	Tricarboxylic acid cycle	not assigned	not assigned	not assigned
13	C0PG78	Monocopper oxidase-like protein SKU5	0.91	Development	not assigned	not assigned	binding
14	A0A1D6M373	Mannosylglycoprotein endo-beta-mannosidase	0.91	Miscellaneous	not assigned	not assigned	not assigned
15	A0A0P0X334	Os07g0176900 protein	0.89	Photosynthesis	not assigned	not assigned	not assigned
16	B4FH75	4-Hydroxy-tetrahydrodipicolinate reductase 2	−0.51	Amino acid metabolism	amine biosynthetic process	cell part	binding
17	B4FAW7	Histidinol-phosphate aminotransferase 2	−0.70	Amino acid metabolism	amine biosynthetic process	not assigned	binding
18	A0A1D6KWD0	Lysine–tRNA ligase	−0.85	Protein	not assigned	not assigned	not assigned
19	A0A1D6LT87	Ras-related protein	−0.89	Signaling	not assigned	not assigned	not assigned
20	O24574	RuBisCO small chain	−1.05	Photosynthesis	biosynthetic process	cell part	carbon-carbon lyase activity
21	Q5I204	Brain acid soluble protein	−1.08	RNA	not assigned	cell part	binding
22	A0A1Q0YQ12	Oil body-associated protein 1B	−1.25	Not assigned	not assigned	not assigned	not assigned
23	A0A1D6I8U5	Flowering time control protein FPA	−1.31	RNA	not assigned	not assigned	not assigned
24	K7UK47	Clathrin interactor EPSIN	−1.69	Not assigned	not assigned	not assigned	not assigned
25	A0A0E0CRT7	Uncharacterized protein	−1.80	Cell	not assigned	not assigned	not assigned
26	A0A0D9XRV3	Adenosylhomocysteinase	−1.88	Amino acid metabolism	not assigned	not assigned	not assigned
27	A0A1D6MQK0	Rab escort protein 1	−1.89	Signaling	not assigned	not assigned	not assigned
28	B6TBI9	Pyridoxamine 5-phosphate oxidase	−1.95	Not assigned	not assigned	not assigned	binding
29	A0A0E0JEZ9	Uncharacterized protein	−1.96	Not assigned	not assigned	not assigned	not assigned
30	B4FTH5	Xyloglucan endotransglucosylase/hydrolase	−2.06	Cell wall	carbohydrate metabolic process	apoplast	catalytic activity
31	B4FZJ2	β-Glucosidase 11	−2.13	Misce	carbohydrate metabolic process	not assigned	catalytic activity
32	B6TUP8	Zinc finger homeodomain protein 1	−2.21	RNA	not assigned	not assigned	binding
33	A0A1D6H8U6	3-Oxoacyl-[acyl-carrier-protein] reductase	−2.31	Lipid metabolism	not assigned	not assigned	not assigned
34	A0A1D6EZ65	Uncharacterized protein	−2.38	Not assigned	not assigned	not assigned	not assigned
35	A0A1D6GLX4	DEK domain-containing chromatin associated protein	−2.54	Not assigned	not assigned	not assigned	not assigned
36	B6TQH7	THA4	−2.63	Not assigned	cellular process	cell part	protein transporter activity
37	C5WUG0	Mitogen-activated protein kinase	−2.75	Signaling	not assigned	not assigned	adenyl nucleotide binding
38	A0A0K9PCU0	RPM1-interacting protein 4	−2.80	Not assigned	not assigned	not assigned	not assigned
39	B6TIG8	Protein arginine N-methyltransferase 1	−2.80	Miscellaneous	cellular macromolecule metabolic process	not assigned	catalytic activity
40	B6TR82	Thioredoxin F-type	−2.81	Redox	biological regulation	not assigned	catalytic activity
41	K3Y2G0	Uncharacterized protein	−2.83	Redox	catabolic process	cell part	not assigned
42	A0A0D3GFF5	Uncharacterized protein	−2.85	Stress	not assigned	not assigned	not assigned
43	B6TYK8	Putative uncharacterized protein	−2.87	Not assigned	not assigned	not assigned	not assigned
44	A0A1D6FY68	SIT4 phosphatase-associated	−2.95	Metal handling	not assigned	not assigned	not assigned
45	A0A1E5V130	Uncharacterized protein	−3.01	Not assigned	not assigned	not assigned	not assigned
46	A0A0N7KSP9	Os11g0247300 protein	−3.18	Cell	not assigned	not assigned	not assigned
47	A0A1D6JR65	Alcohol dehydrogenase-like 2	−3.22	Miscellaneous	not assigned	not assigned	not assigned
48	B6SW97	Putative uncharacterized protein	−3.28	Protein	not assigned	not assigned	not assigned
49	B6U581	Ribosome-like protein	−3.31	Protein	biosynthetic process	cell part	structural constituent of ribosome
50	K4BGM2	Uncharacterized protein	−3.40	Protein	biological regulation	cell part	binding
51	B5QSJ9	Acetyl-coenzyme A carboxylase	−3.53	Lipid metabolism	biosynthetic process	not assigned	acetyl-CoA carboxylase activity
52	A0A1D6L558	Plasmodesmata callose-binding protein 2	−3.67	Miscellaneous	not assigned	not assigned	not assigned
53	A0A0K9NNM0	Cysteine proteinase cathepsin F	−3.69	Protein	not assigned	not assigned	not assigned
54	C0P3K6	Aspartic proteinase A1	−3.75	Protein	lipid metabolic process	not assigned	aspartic-type endopeptidase activity
55	J3LDT9	Uncharacterized protein	−3.76	Not assigned	anatomical structure morphogenesis	cell part	not assigned
56	B9MSV5	Tubulin alpha chain	−3.77	Cell	cellular component assembly	cell part	binding
57	A0A068UVK8	Chlorophyll a-b binding protein	−3.84	Photosynthesis	cellular macromolecule metabolic process	cell part	binding
58	B6SZR1	Chlorophyll a-b binding protein	−3.97	Photosynthesis	cellular macromolecule metabolic process	cell part	binding
59	A0A1D6LJZ2	30S ribosomal protein S16	−4.00	Protein	not assigned	not assigned	not assigned
60	B4FV94	Chlorophyll a-b binding protein	−4.06	Photosynthesis	cellular macromolecule metabolic process	cell part	binding
61	E9KIP1	Photosystem I P700 chlorophyll apoprotein A1	−4.13	Photosynthesis	cellular macromolecule metabolic process	cell part	4 iron, 4 sulfur cluster binding
62	A0A061EVS4	Nascent polypeptide-associated complex beta	−4.23	RNA	biological regulation	not assigned	not assigned
63	A0A1D6IIC3	Nuclear transport factor 2	−4.38	Protein	not assigned	not assigned	not assigned
64	A0A1D5WFY2	Small ubiquitin-related modifier	−4.40	Protein	not assigned	not assigned	not assigned
65	A0A1D6PYA1	60S ribosomal protein L17	−4.61	Protein	not assigned	not assigned	not assigned
66	Q8H6N0	Tubulin beta chain	−4.76	Cell	cellular component assembly	cell part	binding
67	A0A1D5AHD9	RuBisCO large chain	−4.79	Photosynthesis	not assigned	not assigned	not assigned
68	A0A1D1Z067	Elongation factor 1-alpha	−6.21	Protein	not assigned	not assigned	not assigned
69	A0A097PJF2	Structural maintenance of chromosomes protein 1	−9.99	Cell	cell cycle process	cell part	adenyl nucleotide binding

Accession, according to UniProtKB Viridiplantae protein database; Fold change, relative abundance of identified proteins in maize shoot raised from seeds treated with 2000 ppm plant-derived smoke solution; Functional category, protein function categorized using MapMan bin codes. Abbreviations are as follows: cell, cell division/organization/vesicle; transport; CHO, carbohydrate; protein, protein synthesis/degradation/post-translational modification/targeting; RNA, RNA processing/ transcription/binding; Redox, redox homeostasis; DNA, nucleotide binding, and metal ion binding.

## Supplementary Materials

Supplementary materials can be found at https://www.mdpi.com/1422-0067/20/6/1319/s1. Figure S1. GO categories of proteins with differential abundance in maize treated with plant-derived smoke. Figure S2. Metabolic pathway of proteins identified in maize treated with plant-derived smoke.

## Abbreviations

FDR	False discovery rate
LC	Liquid Chromatography
RuBisCO	Ribulose-1,5-bisphosphate carboxylase/oxygenase
MS	Mass Spectrometry
