# Supplementary material for: Molecular Responses of Maize Shoot to a Plant Derived Smoke Solution
Source: Int J Mol Sci. 2019 Mar 15;20(6):1319. doi: 10.3390/ijms20061319 (PMC6471572; doi:10.3390/ijms20061319)
Supplement: Supplementary file 1 [file ijms-20-01319-s001.pdf]

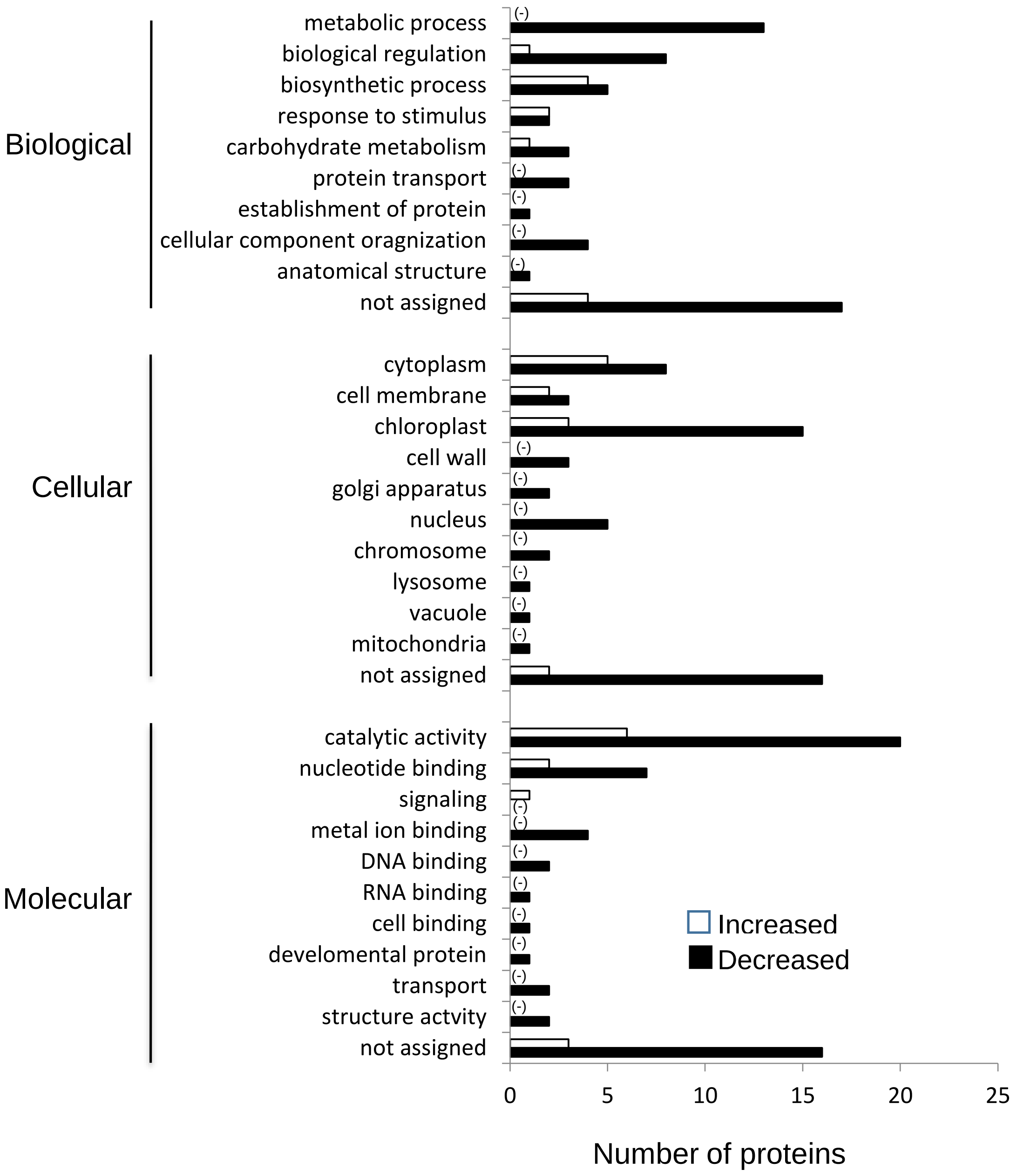

Supplementary Fig. 1

GO categories of proteins with differential abundance in maize treated with plant-derived smoke. Seeds were treated without or with 2000 ppm plant-derived smoke for 4 days. Proteins were extracted from shoot and identified using a gel-free/label-free proteomic technique. Proteins with differential abundance were classified as biological, cellular, and molecular function by WEGO according to the GO terms. A total of 69 proteins identified in maize were analyzed by WEGO. The x-axis indicates the number of proteins. Categories that contained zero proteins are marked with (−).

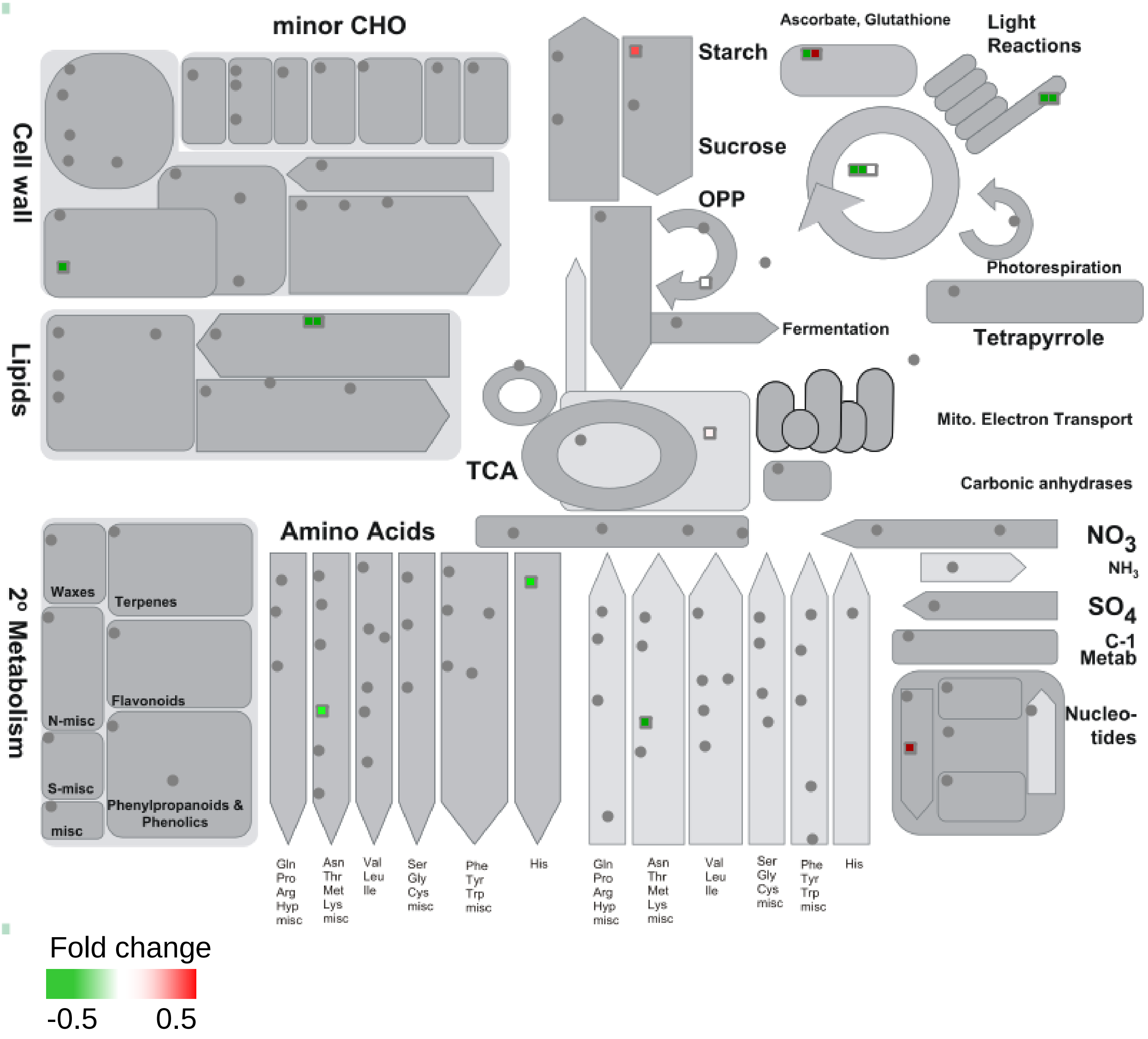

Supplementary Fig. 2

Metabolic pathway of proteins identified in maize treated with plant-derived smoke. Maize seeds were treated without or with 2000 ppm plant-derived smoke for 4 days. The abundance changes of proteins grouped into functional categories related to primary metabolism were visualized using MapMan software. Each square and color indicate the fold change value of a differentially changed protein. Green and red colors indicate a decrease and increase, respectively, in fold change values compared with untreated one. Abbreviations are follows: CHO, carbohydrate; TCA, tricarboxylic acid cycle; OPP, pyrophosphate.
